# A decade and beyond: learnings from HIV programming with underserved and marginalized key populations in Kenya

**DOI:** 10.1002/jia2.25729

**Published:** 2021-06-30

**Authors:** Helgar Musyoki, Parinita Bhattacharjee, Keith Sabin, Ed Ngoksin, Tisha Wheeler, Gina Dallabetta

**Affiliations:** ^1^ National AIDS and STI Control Programme (NASCOP) Ministry of Health Nairobi Kenya; ^2^ Institute of Global Public Health University of Manitoba Winnipeg MB Canada; ^3^ Partners for Health and Development in Africa Nairobi Kenya; ^4^ The Joint United Nations Programme on HIV/AIDS (UNAIDS) Geneva Switzerland; ^5^ Global Fund to fight AIDS, Tuberculosis and Malaria (GFATM) Geneva Switzerland; ^6^ United States Agency for International Development (USAID) Washington DC USA; ^7^ Bill and Melinda Gates Foundation Seattle WA USA

**Keywords:** key populations, scale, 2020 global HIV targets, Kenya, HIV programmes

## Abstract

**Introduction:**

Key populations (KP) continue to account for high HIV incidence globally. Still, prioritization of KP in the national HIV prevention response remains insufficient, leading to their suboptimal access to HIV programmes. This commentary aims to share Kenya’s challenges and successes in achieving 2020 global HIV targets and scaling up the KP programme in the last decade.

**Discussion:**

The KP programme in Kenya has scaled up in the last decade with the inclusion of female sex workers (FSW), men who have sex with men (MSM), people who inject drugs (PWID), transgender people and people in prisons as priority populations in the national HIV response. KP coverage based on official size estimates for FSW is 73%, for MSM is 82%, for PWID through needle syringe programme (NSP) is 71%, and through opioid substitution therapy (OST) is 26% and for transgender people is 5%. The service outcomes for KP have been relatively strong in prevention with high condom use at last paid sex for FSW (92%) and use of sterile equipment among PWID (88%), though condom use at last sex with a non‐regular partner among MSM (78%) is still low. The KP programme has not met care continuum targets for all subpopulations with low case findings. The national KP programme led by the Ministry of Health has scaled up the programme through (a) strategic partnerships with KP‐led and competent organizations, researchers and donors; (b) development of policy guidance and programme standards; (c) continuous sensitization and advocacy to garner support; (d) development of national reporting systems, among others. However, the programme is still struggling with uncertain size estimates; lack of updated bio‐behavioural survey data; inadequate scale‐up of interventions among transgender people and people in prison settings; gaps in reaching adolescent and young KP, and effectively addressing structural barriers like violence and stigma.

**Conclusions:**

To reach the ambitious global HIV targets, sufficient coverage of KP with quality HIV programmes is critical. Despite scaling up the KP programme, Kenya has not yet achieved the 2020 global HIV targets and needs more efforts to scale‐up quality programmes for KP who are underserved in the HIV response.

## 1 INTRODUCTION

New HIV infections in Kenya have fallen from 75,000 among adults in 2010 to 41,416 in 2019 [1]. To continue this trend, the provision of prevention services will increasingly need to be focused on subpopulations with an elevated risk of exposure. In Kenya, the Modes of Transmission (MOT) study in 2009 [2] identified female sex workers (FSW), men who have sex with men (MSM) and people who inject drugs (PWID) as priority populations for the HIV response [3, 4]. In 2020, transgender people and people in prisons and closed settings were also added as priority populations in the national strategic framework [1]. A 2011 integrated bio‐behavioural survey conducted in Kenya showed that HIV prevalence among FSW (29%), MSM (18%; 12.2% for MSM, 26.3% for Male Sex Workers (MSW)) and PWID (19%) was much higher than HIV prevalence among the general population (5.6%) [5, 6]. Another study in Nairobi among transgender people reported an HIV prevalence of 39.9% [7]. The HIV epidemic among key populations (KP) shows geographical and gender diversity with self‐reported HIV prevalence among FSWs ranging from 49% in Homabay to 16% in Mombasa and 36% among female PWID to 17% among male PWID [8]. Newer studies from Nairobi [9] and Mombasa [10] report that HIV prevalence among FSWs decreased over time in all age groups. This decline is attributed by the researchers to the scale‐up of HIV prevention and treatment services in Kenya [9, 10]. Another study conducted with MSM and transgender women in coastal Kenya between 2016 and 17 found that overall, HIV‐1 incidence was 5.1 (95% confidence interval (CI): 2.6 to 9.8) per 100 person‐years (PY) (men who had sex with men exclusively (4.5 per 100 PY)) and transgender women (20.6 per 100 PY) [11].

Recent size estimation exercises, which included virtual mapping for MSM estimated 207,000 FSW, 51,000 MSM, 20,000 PWID and 6,000 transgender people in Kenya [12, 13]. The National AIDS and STI Control Programme (NASCOP) and the National AIDS Control Council (NACC) within the Ministry of Health lead the national KP programme in Kenya [14]. The Government of Kenya and international donors like the US President’s Emergency Fund for AIDS Relief (PEPFAR) and the Global Fund to Fight AIDS, Tuberculosis and Malaria (GFATM) support the KP programme. More than 100 partners implement the programme using a standard HIV combination prevention package [15, 16]. Despite governmental support for the HIV prevention programmes, Kenyan national and county laws continue to criminalize selling sex, same‐sex relationships, drug use and drug possession to date, raising structural barriers for KPs to accessing health services [14].

As a vital member of the Global Prevention Coalition [17], established in 2017 to galvanize greater commitments and investments by countries towards implementing the UNAIDS Global Prevention Roadmap [18], Kenya is committed to achieving the global HIV targets for 2020 [19]. The commentary aims to critically analyse Kenya’s performance against the global targets for KP programme and share learnings from the scale‐up journey in the last decade.

## 2 DISCUSSION

Kenya’s journey with KP programming began in the 1990s with diligent work by researchers and implementers, building a substantial body of evidence to show that KP are critical to the HIV response. KP‐led groups started forming and organizing by early 2000. By the later part of the decade, there was a groundswell of MSM and FSW‐led groups who delivered prevention and treatment services and influenced policy, becoming equal partners in the HIV response. In 2009, using evidence from the MOT study, consistent advocacy led to the inclusion of KP as a priority group in the Kenya National AIDS Strategic Plan (KNASP III 2009‐2014), marking the beginning of government‐led programming developed jointly with KP [20]. Consistent leadership and decisive action at NASCOP and NACC, based on evidence generated by the researchers and implementers, partnerships with the KP communities and donors and successful advocacy with decision makers and political leadership helped scale up the KP programme in Kenya. Significant donor support was provided towards strengthening capacity of KP‐led and other local groups to deliver KP‐competent services as programmes grew. Researchers and implementers enrolled KP in PrEP and vaccine trials and demonstration projects, ensuring the inclusion of their unique needs in the evidence base, to further facilitate scale up of these new technologies.

Table 1 shows Kenya’s progress against 2020 global HIV targets for service coverage and programme outcomes. Kenya has made considerable progress towards achieving the global targets, but large gaps and associated challenges remain. By March 2020, Kenya had expanded programme reach for FSW, MSM, PWID and transgender people to 36, 33, 16 and 3 out of 47 counties respectively. The national KP programme prioritized counties for expansion based on KP size estimates and disease burden. Several counties still need to initiate KP programmes and estimate population sizes to permit rational planning. Size estimates remain an important challenge. Although Kenya used multiple estimation methods like programmatic mapping [21] and virtual mapping [13] to enhance validity, the current population estimate of MSM suggests that only approximately 0.21% of men in Kenya are MSM. The World Health Organization (WHO) and UNAIDS recommend that countries with nationwide estimates of MSM less than 1% should revise their estimates by reviewing the current estimates for biases and, if needed, applying new, more robust methods [22]. As a corollary to the size estimates, PEPFAR set targets to reach 82,000 MSM in Fiscal Year 20 and achieved only 60% coverage.

**Table 1:** Coverage and outcomes of the Kenya key populations programme

Coverage	2020 global targets, %^a^	Progress as on March 2020
1. Female sex workers (FSW)		
1.1 Population size of FSWs^b^		206,609 (129,271 to 206,609)
1.2 Number of FSWs reached with 2 services in past three months^c^	90	15,1070 (73%)
2. Men who have sex with Men (MSM)		
2.1 Population size of MSM^b^		50,556 (30,880 to 50556)
2.2 Number of MSM reached with two services in past three months^c^	90	41,295 (82%)
3. People who inject drugs (PWID)		
3.1 Population size of PWID^b^		19,691 (12,426 to 19,691)
3.2 Number of PWID reached with two services in past three months (OST not included)^c^	90	14,073 (71%)
3.3 PWID who receive OST ^c^	40	5,208 (26%)
4. Transgender People		
4.1 Population size of transgender people^b^		5,783 (2826 to 5783)
4.2 Number of transgender people reached by programmes in the past three months^c^	90	263 (5%)

Outcomes	Global targets 2020, %	Progress as on March 2020, %
1. Female sex workers (FSW)		
1.1 FSW living with HIV who know their status^c^	90	46
1.2 FSW living with HIV who know their status and are on HIV treatment^c^	90	73
1.3 FSW on HIV treatment who are virally suppressed (among those who are eligible and took a viral load test)^c^	90	79
1.4 FSWs who used a condom with last client^d^	95	92
1.5 FSW who experienced police violence in the last six month^d^		48
2. Men who have sex with Men (MSM)		
2.1 MSM living with HIV who know their status^c^	90	52
2.2 MSM living with HIV who know their status and are on HIV treatment^c^	90	80
2.3 MSM on HIV treatment who are virally suppressed (among those who are eligible and took a viral load test)^c^	90	74
2.4 MSM who used a condom at last anal sex^d^	90	79
2.5 MSM who experienced police violence in the last six months^d^		20
3. People who inject drugs		
3.1 PWID living with HIV who know their status^c^	90	43
3.2 PWID living with HIV who know their status and are on HIV treatment^c^	90	68
3.3 PWID on HIV treatment who are virally suppressed (among those who are eligible and took a viral load test)^c^	90	64
3.4 PWID who used safe injecting equipment during last injection^d^	95	88
3.5 PWID who experienced drug overdose in the last six months^d^		40
3.6 PWID who experienced police violence in last six months^d^		44

ART, Anti‐retroviral therapy; FSW, Female sex workers; MSM, men who have sex with men; NSP, Needle syringe programme; OST, Opioid substitution therapy; PWID, people who inject drugs.

^a^ The HIV Prevention Revolution Roadmap of UNAIDS recommends programmatic coverage and outcome targets for the KP program [23]. Along with a target of 90% prevention program coverage of key populations, the other prevention outcome targets to be achieved by 2020 were (i) condoms and safe behaviours: (a) 95% condom use at last paid sex for FSW; (b) 90% condom use at last sex (targets were set before PrEP was available) with a non‐regular male partner for MSM; (ii) access to treatment continuum a) 90% of the KPs aware of their HIV status; b) 90% of the diagnosed people living with HIV receive ART; (c) 90% of PLHIV on ART are virally suppressed and (iii) access to comprehensive harm reduction services: (a) 40% of people who inject(ed) opioids use opioid substitution therapy (OST) (b) 95% sterile injecting equipment use at last injection for PWID.

^b^ data from Key Population Size Estimation, 2018.

^c^ data from reports submitted by all implementing partners to NASCOP on a quarterly basis for the quarter January to March 2020.

^d^ data from annual population based behavioural survey conducted by NASCOP using polling booth survey methods. The last survey was conducted in 2017.

In the first quarter of 2020, against the current official population size estimates, programme coverage (defined as reached with two services in the past three months) was 73% for FSWs, 82% for MSM, 71% for PWID (one service being needle and syringe programme) and 5% for transgender people. Kenya scaled‐up OST services in the last five years by initiating 9 Medically Assisted Treatment (MAT) clinics and had enrolled 5208 PWID (26% of estimated PWID). Enrolment of opioid users in the Opioid Substitution Therapy (OST) programme fell short of the 40% target. This suggests that programmes need further expansion. There was also no data available for people in prison settings at the time of writing this commentary. The KP programme must scale up programmes for transgender people, by adopting the national guidelines [24] and for people in prison settings in partnership with National Prison Services, with urgency.

The behavioural programme outcomes, measured by a 2017 population‐based survey, show condom use at last sex with a client for FSW was 92% and at last sex for MSM was 79%. The KP programmes needs to prioritize access and utilization of prevention services (condoms and PrEP) among MSM. Eighty‐eight percent of PWID reported using safe injecting equipment during the last injection; unfortunately, 40% also reported experiencing a drug overdose. Opioid overdose, a leading cause of mortality amongst PWID [25], remains high in Kenya and hence improving access to naloxone at the community level is essential.

The treatment programme outcomes showed that 46% of FSW living with HIV knew their HIV status, 73% of all FSW living with HIV were receiving ART and 79% of all FSW receiving ART demonstrated viral suppression; the similar outcome for MSM was 52% to 80% to 74% and PWID were 43% to 68% to 64%. It is concerning that achievement of care continuum targets among KP, especially the first 90, is much lower than Kenya's general population (80% to 96% to 91%) [26]. The case‐finding rates have remained steady between 1% to 1.5%, probably because the programme is still not reaching specific high‐risk subpopulations. Considering the country's geographic diversity, it would be important to assess the at‐risk KP subpopulations missed by the testing and treatment programmes like the adolescent and young KP who constitute 9% to 12% of the estimated KP [12, 27]. Kenya also needs to develop KP differentiated care models linking community outreach and clinical efforts and scaling up community ART initiation and dispensation for KP to address the gaps related to the second 90 target.

In terms of structural programme outcomes, a high proportion of KP reported experiencing police violence in the last six months: FSW (48%), MSM (20%) and PWID (44%) in the population‐based survey. Although reporting of violence and support provided in response to the reports by implementing partners has increased in the last decade [14], police violence against KP remains high. Committed funding sources to scale‐up interventions to address human rights‐ and gender‐related barriers to services for KP in a comprehensive and coordinated way is needed.

While KP programme scale‐up has been a priority for donors, there is disagreement on prevention programme priorities and approaches, with PEPFAR programmes focusing largely on case finding and treatment linkage for KP rather than comprehensive prevention. Kenya has not had a KP bio‐behavioural survey since 2011 as there has been no consensus on a protocol and method. This has posed challenges in measuring the KP programme outcomes and impact [28]. The complete national KP programme data has still not moved to an electronic database raising the need to invest in real‐time data systems. The KP programme also does not have a standard unit cost to advocate for adequate resources from donors and explore sustainable domestic funding options. The HIV prevention shadow report for Kenya developed by civil society in 2020 recommends the need for (a) improved leadership especially at county level (b) removal of legal barriers (c) increased investment in structural interventions and (d) enhanced access to new prevention technology for KP [29].

Despite existing gaps, there are several learnings that emerge from the Kenya KP programme. Some of the successful strategies that provided confidence to donors, implementers and researchers to participate in the scale‐up plan in Kenya include, (a) formation of the KP Technical Working Group (TWG); (b) development of policy guidance and programme standards; (c) decentralization of the response at county level; (d) setting up a robust monitoring system with defined targets from grassroots to national level**;** (e) continuous advocacy and sensitization of service providers, stakeholders and decision makers; (f) development of a Technical Support Unit to support scale‐up**;** (g) establishment of diverse models of services provision including “ one stop shop” and integrated models; (h) strategic partnership with a variety of stakeholders including KP led organizations and KP research advisory groups (like G10); (i) active promotion of KP led service delivery models with support to more than 25 KP led organizations and (j) proactive documentation to create visibility for the programme and the populations. Some of the key strategies are described below.

The KP Technical Working Group (TWG) is a formal coordination body at the national level, bringing donors, researchers, implementers and KP‐led organizations with government partners to engage in collective decision making and shared responsibility [16]. The group has been meeting every quarter for the last eight years to discuss the KP programme's critical technical and management issues. Decisions related to geographical demarcations for programming, annual targets, programme gaps are made in this forum. The TWG have emerged as a safe space in recent years to seek accountability and engage in joint solutioning. In the last five years, these forums have been decentralized at the county level, where stakeholders engage quarterly, review their local programme and devise local solutions. Development of policy guidance early in the programme scale‐up phase [30] and setting standards and operating procedures for the KP programme helped provide an enabling environment and maintain quality and consistency during the scale‐up phase [16, 27, 31]. As addressing legal barriers through law change takes time, the national KP programme prioritized developing a conducive policy environment to ensure that constitutional rights of KP to access the highest quality health care are protected. The policy guidance provided recognition to the programmes targeting KP and protection to the implementers to scale‐up programmes even when behaviours related to KP are criminalized [32]. The guidelines and standard operating procedures guided donors and implementers to follow a common minimum standard and approach to KP programming. The KP TWG has been responsible for developing these standards and operating procedures, allowing stakeholders to participate and own the process. KP have played a key role in these processes, ensuring that the guidelines are sensitive to their lived realities and address their specific and diverse needs. Setting up a robust monitoring system that defined standard data collection and reporting tools to measure and monitor programme interaction by individual KP and site [33]. This helped the KP programme generate sub‐county, county, national reports and cascades to monitor progress across biomedical, behavioural and structural interventions [34] early on in the scale‐up phase. The data collection for routine monitoring involve different cadres of programme staff, including peer educators. The population size estimates, generated every five years, provide information to set the programme targets. Programme data are analysed every quarter to assess programme progress, using these targets as denominators. The use of KP programme data has become a norm now in KP programming at all levels; from a peer educator to a national programme manager [34]. The national KP programme also conducts population‐based outcome surveys to measure KP programme outcomes for behavioural and structural interventions. These surveys use polling booth survey method [35] and involve the KP community as researchers [36]. Efforts are on to move this data collection and compilation system into an electronic platform to increase the ease of individualized programme tracking and data use.

In an environment where behaviours of KP are criminalized and judged based on prevailing norms around gender, identity, sexuality and drug use, it has been an uphill task to scaling up KP programme and maintain the fidelity and quality of the programmes. During this journey of one decade, the KP programme has experienced several setbacks, including the attack on MSM clinics in Kilifi; community agitation against the needle and syringe programme and death of PWID due to heroin overdose crisis caused by the shortage of supply. One such example has been the impact of the President of Uganda signing the Anti‐Homosexuality Bill in 2014 on the KP programme in Kenya. Within a month of signing the bill, a cross‐party parliamentary group was set up in Kenya demanding stronger laws against homosexuality [37]. This act of Kenyan parliamentarians facilitated public rallies and threats against gay and lesbian organizations, creating panic and fear among MSM groups. MSM led organizations had to suspend their services and close their offices for several weeks. The MSM clients also stayed away from services to avoid contact with people fearing hostility. The national KP programme guided by the TWG brought together stakeholders, including UN bodies and KP community groups, to strategically address the issue. A multipronged strategy was developed which included (a) convincing the Health Minister to issue a statement emphasizing the need to protect the health rights of MSM and supporting the organizations providing HIV services under the national HIV response; (b) supporting and linking the MSM groups in Kenya with the global advocacy against criminalization and legal barriers to develop community‐led advocacy plans; (c) strengthening the crisis response system to support the MSM community in case of violence or backlash against them; (d) advocating with the parliamentarians and sensitizing them on HIV prevention work with KP and (e) providing support to the implementing partners to provide services to the MSM ensuring their safety. In Kenya, non‐confrontational advocacy and negotiation by civil society organizations to adopt policies or practices have been effective strategies to create an enabling environment for scaling up programmes for KP [32]

The KP programme's future priorities include increasing its scale and coverage and ensuring 2020 global outcome targets are achieved in the next one to two years while working towards the imminent 2025 targets [38].

## 3 CONCLUSIONS

Achieving the ambitious global impact targets of reducing new infections requires sufficient coverage of KP with quality HIV programmes [39]. Although Kenya did not achieve all global targets for KP programmes for 2020, gaps and priorities have been identified and plans are in development to close them. Consistent leadership at the national level and strategic partnership with implementers, KP community, researchers and donors have supported the scale‐up journey and will need to continue in the future. The leadership of KP‐led networks in implementation and advocacy contributed to positive change in policy and practice. The establishment of robust management and monitoring systems and a mechanism to coordinate and take collective responsibility to address challenges will help achieve quality HIV programmes for underserved KP in Kenya.

## Competing interests

The authors declare no competing interests.

## Authors’ contributions

HM and PB conceptualized the paper. PB designed the plan of analysis. HM and PB wrote the first draft of the paper with additions and edits from GD. KS, EN and TW reviewed the paper and made additions and edits. All authors have read and approved the final manuscript.

## Abbreviations

FSW, Female Sex Workers; GFATM, Global Fund to Fight AIDS Tuberculosis and Malaria; KP, key populations; MAT, Medically Assisted Therapy; MSM, Men who have sex with men; NACC, National AIDS Control Council; NASCOP, National AIDS and STI Control Programme; OST, Opioid Substitution Therapy; PEPFAR, President's Emergency Plan for AIDS Relief; PWID, People who Inject drugs; TSU, Technical Support Unit; TWG, Technical Working Group.
